# Lack of redundancy between electrophysiological measures of long-range neuronal communication

**DOI:** 10.1186/s12915-021-00950-4

**Published:** 2021-02-08

**Authors:** Daniel Strahnen, Sampath K. T. Kapanaiah, Alexei M. Bygrave, Dennis Kätzel

**Affiliations:** 1 Institute of Applied Physiology, Ulm University, Albert-Einstein-Allee 11, 89081 Ulm, Germany; 2 Department of Neuroscience, Johns Hopkins University, Baltimore, USA

**Keywords:** Hippocampal-prefrontal coherence, wPLI, Phase-amplitude coupling, Phase-locking value, Pairwise phase-consistency, Spike-phase coupling, Granger causality, Partial directed coherence, Amplitude cross-correlation, *Gria1*

## Abstract

**Background:**

Communication between brain areas has been implicated in a wide range of cognitive and emotive functions and is impaired in numerous mental disorders. In rodent models, various metrics have been used to quantify inter-regional neuronal communication. However, in individual studies, typically, only very few measures of coupling are reported and, hence, redundancy across such indicators is implicitly assumed.

**Results:**

In order to test this assumption, we here comparatively assessed a broad range of directional and non-directional metrics like coherence, Weighted Phase Lag Index (wPLI), phase-locking value (PLV), pairwise phase consistency (PPC), parametric and non-parametric Granger causality (GC), partial directed coherence (PDC), directed transfer function (DTF), spike-phase coupling (SPC), cross-regional phase-amplitude coupling, amplitude cross-correlations and others. We applied these analyses to simultaneous field recordings from the prefrontal cortex and the ventral and dorsal hippocampus in the schizophrenia-related *Gria1-*knockout mouse model which displays a robust novelty-induced hyperconnectivity phenotype. Using the detectability of coupling deficits in *Gria1*^*−/−*^ mice and bivariate correlations within animals as criteria, we found that across such measures, there is a considerable lack of functional redundancy. Except for three pairwise correlations—PLV with PPC, PDC with DTF and parametric with non-parametric Granger causality—almost none of the analysed metrics consistently co-varied with any of the other measures across the three connections and two genotypes analysed. Notable exceptions to this were the correlation of coherence with PPC and PLV that was found in most cases, and partial correspondence between these three measures and Granger causality. Perhaps most surprisingly, partial directed coherence and Granger causality—sometimes regarded as equivalent measures of directed influence—diverged profoundly. Also, amplitude cross-correlation, spike-phase coupling and theta-gamma phase-amplitude coupling each yielded distinct results compared to all other metrics.

**Conclusions:**

Our analysis highlights the difficulty of quantifying real correlates of inter-regional information transfer, underscores the need to assess multiple coupling measures and provides some guidelines which metrics to choose for a comprehensive, yet non-redundant characterization of functional connectivity.

**Supplementary Information:**

The online version contains supplementary material available at 10.1186/s12915-021-00950-4.

## Background

Communication between different brain regions is vital for cognition and emotion and is impaired in a variety of neurological and psychiatric disorders, including schizophrenia and depression. In order to better understand interregional communication in health and disease at the electrophysiological level in rodent models, local field potentials (LFPs) and sometimes action potentials (spikes) are typically recorded from two or more brain areas simultaneously in awake subjects. Subsequently, some measure of interdependency of the signals from two regions are computed (see Table 1 for an overview).

**Table 1:** Common measures of synchrony and directionality in neuronal communication

Category	Acronym	Metric	Description	References
Non-directed coupling, synchrony	–	Coherence (magnitude)	Magnitude of the complex cross-spectrum	[1, 2]
	ImC	Imaginary part of coherence	Discards the real component of the cross-spectrum	[3]
	PLI	Phase Lag Index	Disregards the magnitude of the cross-spectrum and averages the sign of phase differences	[4]
	wPLI	Weighted phase lag index	Phase lags are weighed by the magnitude of the imaginary component of the cross-spectrum	[5]
	PLV	Phase-locking value	Circular resultant vector length of the phase differences	[6]
	PPC	Pairwise phase consistency	Computed based on the distribution of phase differences	[7]
Directed (lead/lag, LFP-based)	–	Coherence phase angle	Angle of the complex cross-spectrum	[8, 9]
	CC	Cross-amplitude coupling, amplitude cross-correlation	Instantaneous amplitudes of two filtered LFPs are cross-correlated and the lag at which the peak occurred is determined	[10]
Directed (causal influence)	GC	Granger causality	Quantifies if the past of one time series can predict the future of another time series using autoregressive modelling	[11–14]
	npGC	Non-parametric Granger causality	Granger causality based on spectral matrix factorization	[15]
	PDC	Partial directed coherence	Normalized metric based on GC that measures direct influence from one time series to another	[16]
	DTF	Direct transfer function	Adaptation to multiple input variables closely related to PDC	[17, 18]
Directed (phase-locking of local activity)	SPC, MRL	Spike-phase coupling, Mean resultant vector length	Circular concentration of the phase distribution at which spikes occurred	[2, 19–21]
	PAC, CFC, MI	Phase amplitude coupling, cross-frequency coupling, modulation index	Modulation of the amplitude of high-frequency oscillations in one area by the phase of low-frequency oscillations from another area	[22–24]
Directed (lead lag, spike-based)	–	Phase angle of MRL	Mean phase at which spikes occurred	[20]
	–	Phase-shifted MRL	Calculation of the MRL based on phases at shifted lags	[2, 19]

For example, an influential hypothesis known as *communication through coherence* (CTC) states that information exchange between two connected brain areas depends on the timing of the arrival of incoming activity in a specific phase of a certain network oscillation [25–28]. Therefore, coherence measures a synchrony of oscillations in a certain frequency range and with a certain phase shift that may allow the activity generated in one region to optimally affect the activity of another region.

In general, measures of phase synchronization aim to determine if two signals have a consistent phase relationship between each other. Despite being widely used, coherence is prone to confound by volume conduction [22, 29]. Therefore, alternative measures of phase synchronization have been suggested. Nolte et al. demonstrated that using only the *imaginary component of coherence* (ImC) effectively reduces the influence of a volume-conducted signal originating from a common source [3]. Alternatively, the *Phase Lag Index* (PLI) may be used, which disregards the magnitude of the phase lag between signals from two brain regions but evaluates if they differ from a symmetrical distribution [4]. The weighted PLI (wPLI), in turn, applies the combined advantages of the ImC and PLI by taking the detected phase lead or lag and weighing it by the magnitude of the ImC [5]. A constraint related to measures of phase synchronization like the ImC, PLI and wPLI is sample size bias, i.e. the observation of spurious non-zero synchrony even in the absence of real connections which increases with a lower number of samples [5]. Therefore Vinck et al. additionally introduced a debiassed estimator of the wPLI which is more independent from sample size and thus has a higher statistical power than previous measures [5]. For the sake of clarity, the debiassed wPLI will be referred to as wPLI throughout this study, and the stated older measures are not used.

It should be noted that several other metrics for phase synchronization exist, such as the phase-locking value (PLV) [6] and pairwise phase consistency (PPC) [7]. These measure the constancy of the difference between the instantaneous phases of two signals obtained either by applying Hilbert, wavelet or Fourier transformation and quantifying the distribution of phase differences either by taking the vector average or by determining the distribution of phase differences across observations, respectively. While PPC and PLV are very similar measures, the main advantage of the PPC metric is that it is not biassed by sample size and therefore more suitable for comparing datasets with varying sample size as reviewed in [30].

The stated measures of phase synchronization are attempts to quantify *non-directed* connectivity. This means that the quantification of coupling is essentially based on correlation analysis, ignores its temporal structure and assumes no direction of the influence from one region to another [30, 31]. However, LFP data can also be used to measure *effective* or *directed connectivity* [31]. These are parameters that quantify the potentially causal influence that the activity in one region exerts on another region by taking recurring pairwise patterns in the time series obtained from both regions into account. A computationally simple measure to detect directionality between two time series is *cross-correlation*. That means that correlations are calculated as the LFP signals are incrementally shifted against one another to obtain a cross-correlation as function of temporal shifts (lags). Adhikari et al. developed a method termed *amplitude cross-correlation* or *cross-amplitude coupling* in which the instantaneous amplitudes of two oscillatory signals filtered in a certain frequency range are cross-correlated to determine if one is leading or lagging the other [10]. If the lags at which the peak of the amplitude cross-correlation function occurs are significantly different from 0 ms, it is indicated that one region leads the other one with a certain consistency, which could be due to a directional influence from the leading onto the lagging region. This method was able to identify directional connectivity in the brain related to working memory and fear processing [32–34].

A different measure of directed influence is *Granger causality* (GC). It aims to infer causation based on the notion that one signal is helpful in predicting the other. In *parametric* GC, two separate autoregressive models (ARMs) are calculated and statistically compared: a univariate ARM, where the signal is predicted by a weighted combination of its own past values, and a bivariate ARM where the signal is additionally predicted by the second signal. If the inclusion of the bivariate AR leads to a reduction of variance of the predicted signal, one signal is said to Granger-cause the other [11]. GC can also be computed with *non-parametric* methods where the same information is obtained by first calculating the cross-spectral density matrix and then applying Wilson’s spectral matrix factorization as input to the GC algorithm; this approach has been demonstrated to be equivalent to parametric GC [35]. The mathematical foundations of GC and its application to neuroscience have been reviewed extensively elsewhere [11–14]. Related measures that can either be based on *multivariate* autoregressive models or on non-parametric methods for directionality estimation and allow analysis of more than two channels include the *directed transfer function* (DTF) [17] and *partial directed coherence* (PDC) [16]; see [18] for a review.

Other indicators of inter-regional communication that partly circumvent problems caused by volume conductance and are typically interpreted as indicating a causal directional influence include those that measure *different types* of neuronal activity in the different regions, i.e. a low-frequency LFP oscillation (usually in the theta-range) in the presumed dominating region and a local and high-frequency activity at the receiving end. In contrast to the metrics introduced before, historically, such measures were introduced by way of an actual *biological* discovery of such coupling phenomena, rather than by a priori mathematical considerations on how to best assess inter-regional communication. One option is to quantify the extent to which oscillations of *distinct* frequencies are coupled to each other, a phenomenon called cross-frequency coupling (CFC, [36]). Particularly, local phase-amplitude coupling (PAC)—the statistical relationship between the phase of a low-frequency and the amplitude of a high-frequency component—plays an important role in memory processing in the hippocampus of rats [37, 38] and humans [39]. However, *cross-regional* PAC between the hippocampus and prefrontal cortex has also been used and was associated with directed information flow and cognitive functions [22–24, 40]. Since high-frequency brain oscillations mainly reflect local aspects of information processing and low-frequency brain rhythms are relevant for inter-regional communication, CFC might represent a mechanism of transferring information from large-scale neuronal networks to local processes [36, 41].

Another widely used measure is based on the recording of spikes in one (potentially the *influenced*) region alongside the LFP in another (potentially the *influencing*) region. Spikes are generally not considered to be confounded by volume conductance or referencing, and they represent a more direct readout of the actual neuronal activity of a region. Phase-locking of neuronal firing to theta frequency hippocampal oscillations was shown for example in the prefrontal cortex (PFC) [1, 19], entorhinal cortex [42] and the amygdala [43]. For example, action potentials in these brain regions occurred rhythmically at the same phase of the hippocampal theta rhythm. Such spike-phase coupling (SPC) was observed to correlate with performance in multiple cognitive tasks [1, 19, 44] and has been used to evaluate coupling deficits in mouse models related to schizophrenia [2, 20, 45].

The above-mentioned measures have been widely used for two decades to assess inter-regional neuronal communication in rodents during a variety of cognitive tasks and disease-related manipulations, mostly involving recordings from the hippocampus and prefrontal cortex [1, 2, 20, 21], but also increasingly from the thalamus [46] and the amygdala [47]. However, typically, only one or two measures of coupling are calculated and interpreted as sufficient surrogate to quantify task- or manipulation-related differences in actual information exchange between the analysed regions. In this analytical set-up, the contingency of the achieved conclusions on the choice of the coupling measure is usually not evaluated, but the redundancy of the various measures is implicitly assumed. This assumption is not justified, however, given the mathematical and partly biological differences between these constructs. Likewise, the dependence of the conclusions on the exact placements of electrodes within the analysed regions and the choice of reference are often not evaluated either. This presents a problem especially when interpreting negative data, i.e. the supposed absence of differences in coupling.

We therefore sought to evaluate the redundancy and contingencies of such coupling metrics. To this end, we recorded data during a simple behavioural assay—novelty-induced locomotion and its habituation over time—in *Gria1*^−/−^ (KO, knockout) mice and their littermate controls. We have recently shown that the *Gria1*-KO model, which recapitulates some behavioural deficits relevant to schizophrenia, shows profound and state-dependent aberrations of hippocampal-prefrontal coupling in this task [48]. We focused on the most widely used connectivity measures—coherence magnitude and phase angle, wPLI, PPC, PLV, cross-amplitude coupling, parametric and non-parametric GC, PDC, DTF, cross-regional PAC, SPC and SPC-related directionality—with respect to three ‘litmus tests’ for redundancy: (a) detection of KO-related alterations of coupling across the 10 min test, (b) detection of KO-related changes of a measure over time and (c) bivariate within-animal correlation of the analysed measures. We investigated connectivity between the medial prefrontal cortex (PFC) and the hippocampus—both the dorsal (dHC) and the ventral (vHC) partition. For the majority of the analysis, four commonly used frequency bands, delta (*δ*, 1–4 Hz), theta (*θ*, 5–12 Hz), beta (*β*, 15–30 Hz) and low gamma (*γ*, 30–48 Hz) are distinguished, whereby the analysis of theta and gamma may be regarded as particularly informative due to the existence of spectral peaks indicating real underlying oscillatory processes.

## Results

### Elevated locomotor activity in *Gria1*-KO mice during measurement of interregional communication

**Fig. 1:**
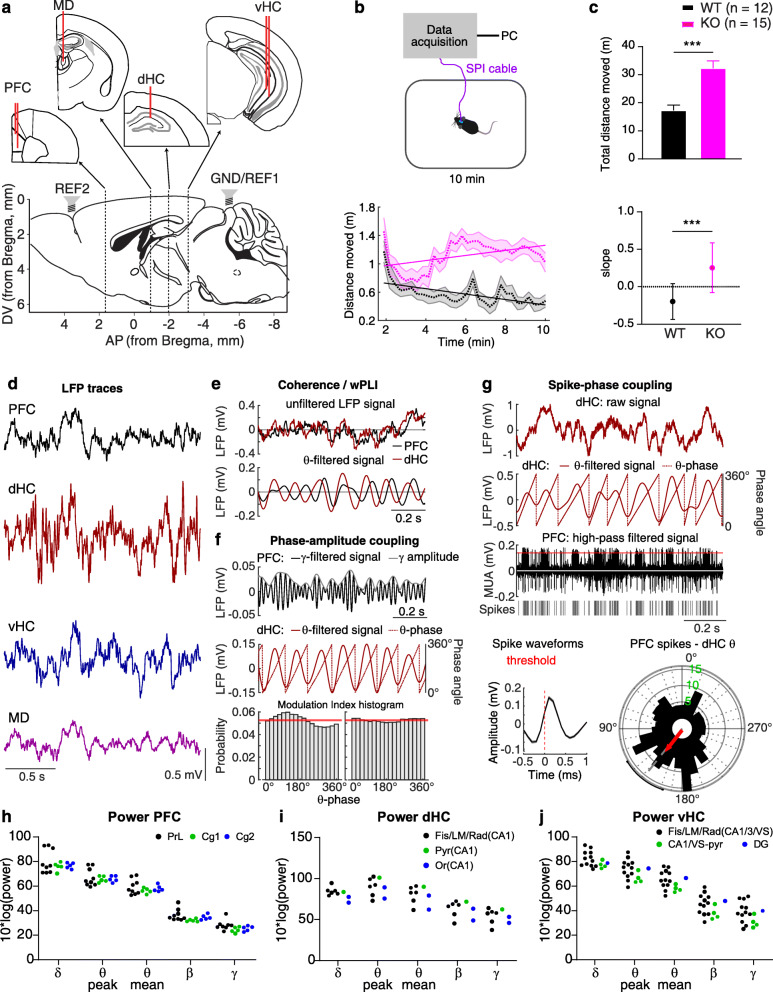
Experimental set-up, behaviour and recorded signals. **a** Placement of LFP and screw electrodes. **b** Top, experimental set-up; bottom, distance moved in 20 s bins by *Gria1*^−/−^ (KO, purple) and wild-type controls (WT, black); dashed line indicating mean; shaded region representing SEM; solid overlaid line representing linear interpolations across time. **c** The same data as in **b** but displayed as total distance moved in 10 min (top) and slope of the interpolated line (bottom). ***p < 0.001, *t* test. **d** Examples of unfiltered LFP traces recorded in the four brain regions. **e** Illustration of the processing for connectivity measures using the same LFP frequency band in both regions; raw LFP signal (top) and LFP signal filtered in a specific frequency range (bottom). **f** Illustration of cross-regional θ-γ PAC, whereby the signal in one region is filtered in the low-γ range and the amplitude is extracted (top), while the signal in the other region is filtered in the θ range and Hilbert-transformed to extract the θ phase (middle). The coupling strength is derived as a modulation index (MI) measuring the phase-related change of γ amplitude (bottom). **g** Illustration of SPC; the hippocampal LFP (top) is filtered in the theta range and the instantaneous phase angle is extracted by linear interpolation (below, brown); the prefrontal high-pass filtered signal reveals MUA from which spikes are extracted by thresholding (below, single spikes, and bottom left, average of all extracted PFC spikes, black). A circular histogram is computed by assigning each spike to its theta phase angle, and the average of all vectors is calculated as mean resultant vector (red) whose length (MRL) is taken as indicator of SPC strength (bottom right). **h**–**j** Power of LFP in the indicated frequency bands (*x*-axis) and region (top of panel) displayed for each individual electrode that contributed to the WT dataset colour-coded by the sub-division in which it was placed; hippocampal layers: pyramidal (Pyr), stratum oriens (Or), lacunosum-moleculare (LM), radiatum (Rad), and fissure (Fis). No statistical analysis was done given that rare placements

In order to measure inter-regional coupling, we implanted 15 adult *Gria1*^−/−^ mice and 12 littermate controls unilaterally with LFP electrodes in 4 regions, PFC (2 electrodes), mediodorsal thalamus (MD, 1 electrode), dHC (1 electrode) and vHC (2 electrodes), and inserted screws for ground and reference above the cerebellum and frontal cortex, respectively (Fig. 1a). Recordings from all sites were made during a 10-min test of novelty-induced locomotor activity which confirmed the strongly elevated behavioural activity and failure of its short-term habituation over time in *Gria1*^−/−^ mice, as observed before (Fig. 1b, c [48]). After the experiments were completed, the placement of electrodes was evaluated through electrolytic lesion sites, and misplaced electrodes were excluded from the dataset; data from the MD was disregarded for most of the subsequent analysis because of the low number of animals with accurate placements. In accordance with our previous study in this mouse line [48], we recorded and analysed all data as referenced to the ground screw above the cerebellum by default and used the data from the frontal reference screw for a separate analysis (displayed in Fig. 7). We extracted LFP signals (Fig. 1d, e) from all depth electrodes and multi-unit activity (MUA) spikes from the prefrontal wires. For PAC, amplitude cross-correlations and SPC, the theta phase angle was extracted using a Hilbert transform or linear interpolation between consecutive cycles (Fig. 1f, g). Additionally, we sorted the LFP power values obtained from each electrode in distinct frequency bands according to the placement of electrodes in different subdivisions of the PFC (PrL, Cg1 and Cg2), dHC (apical dendritic layers of CA1, CA1 pyramidal cells, CA1 stratum oriens) and vHC (apical dendritic layers of CA1/CA3, CA1 pyramidal cells, dentate gyrus). While we did not conduct statistical analysis given the much smaller number of sites outside the target region (PrL in PFC and apical dendritic layers, including fissure, in the hippocampus), a qualitative inspection suggested that the placements inferred from lesion sites did not noticeably alter the obtained spectral LFP power (Fig. 1h–j).

### Differences in detecting delta and gamma-range coupling in *Gria1*-KO mice across measures of synchrony

**Fig. 2:**
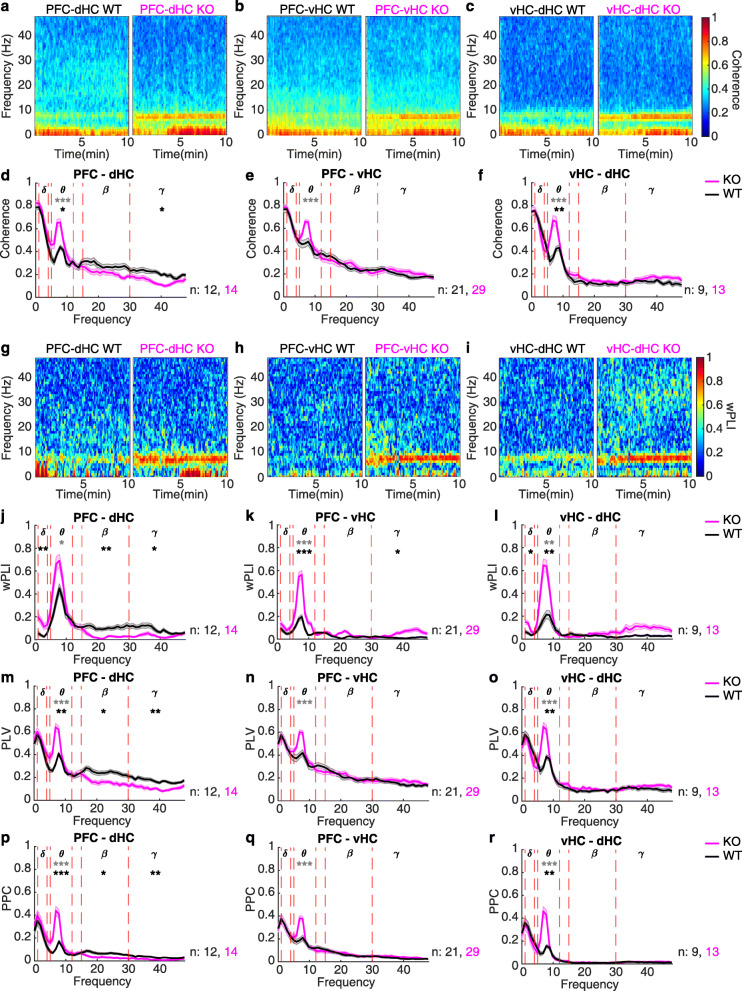
Non-directional measures of synchrony in *Gria1*^−/−^ and wild-type controls across 10 min novelty-induced activity. **a**–**l** Spectrogrammes (**a**–**c**, **g**–**r**) and frequency spectra (**d**–**f**, **j**–**r**) displaying coherence (**a**–**f**), wPLI (**g**–**l**), PLV (**m**–**o**) and PPC (**p**–**r**) along the PFC-dHC (**a**, **d**, **g**, **j**, **m**, **p**), PFC-vHC (**b**, **e**, **h**, **k**, **n**, **q**) and vHC-dHC (**c**, **f**, **i**, **l**, **o**, **r**) connections. Dotted red lines in spectra indicate boundaries of the analysed frequency bands named by the greek letters at the top. Stars indicate significant differences between genotypes (*t* test) in mean (black) or peak (grey) synchrony metrics. Lines display mean ± SEM. *p < 0.05; **p < 0.01; ***p ≤ 0.001

We first analysed phase synchronization along the two prefrontal-hippocampal connections (PFC-dHC and PFC-vHC) and within the hippocampus (vHC-dHC) using coherence, wPLI, PLV and PPC (Fig. 2a–r). We confirmed our previous observation [48] that PFC-dHC theta coherence is strongly elevated in *Gria1*-knockouts in a novel environment and further increases with time, mirroring the spatial exploration behaviour of this genotype (Fig. 1b, c, Fig. 2a, d). However, this phenotype was by no means specific to the PFC-dHC coupling, but also re-appeared in the PFC-vHC and vHC-dHC connections suggesting a broader deficit of excessive theta-range connectivity (Fig. 2b, c, e, f). Reassuringly, the same phenotype was revealed by the wPLI, PLV and PPC metric across connections (Fig. 2g–r). However, when inspecting the other frequency bands, findings were not particularly consistent between wPLI and the other three measures (which appeared very similar to each other). While all indicators revealed a reduced gamma-range PFC-dHC coupling in knockouts, a sole analysis with wPLI suggested further differences in the delta (PFC-dHC, vHC-dHC) and gamma (PFC-vHC) ranges that would have gone undetected, if using the other metrics (Fig. 2d–f, j–r). Also, qualitatively, wPLI resulted in spectra with a quite different shape compared to the other ones.

### Differences in detecting elevated inter-regional theta-range coupling in *Gria1*-KO mice across measures of directional communication

**Fig. 3.**
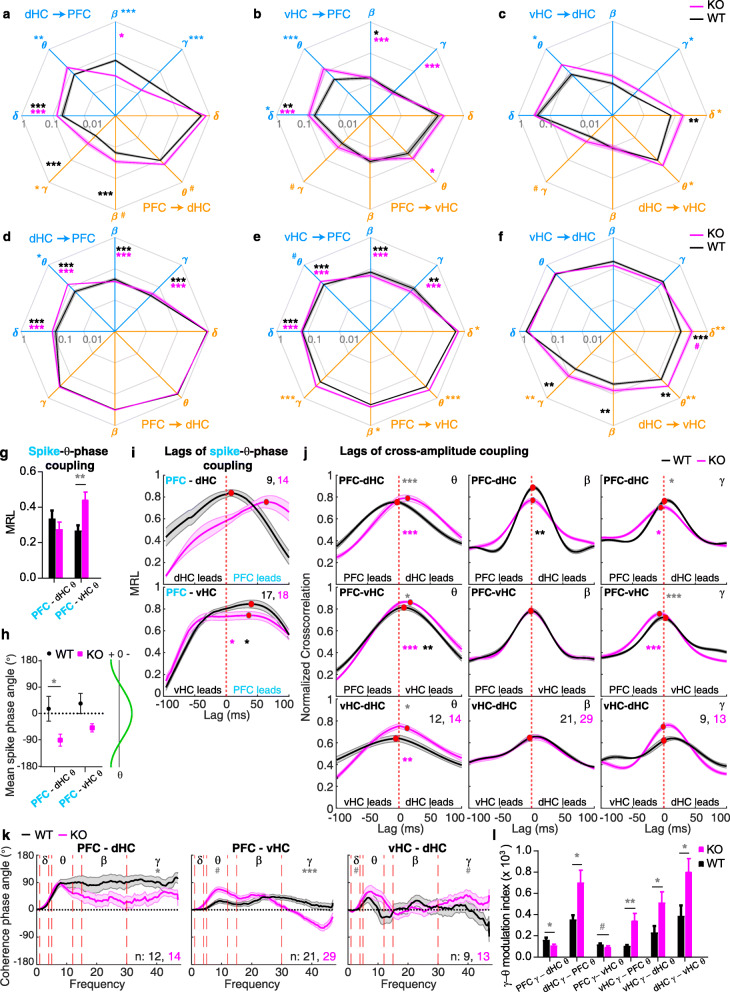
Directional metrics of inter-regional coupling in *Gria1*^−/−^ and wild-type controls across 10 min novelty-induced activity. **a**–**c** Parametric GC on log_10_ scale in the frequency bands indicated by greek letters and along the directional connections identified by the colour (blue: dHC➔PFC (**a**), vHC➔PFC (**b**), vHC➔dHC (**c**); orange: reverse of the before). Statistical indicators in the same colour identify a difference between genotypes (Sidak); statistical indictors in black (WT) or purple (KO) refer to a significant difference between the GC values of the two opposing directions within the colour-coded genotype whereby the location of the indicator identifies the direction with *smaller* average GC. **d**–**f** The same display as **a**–**c** but for PDC. **g** Mean resultant vector length (MRL) as an indicator of SPC of prefrontal spikes to hippocampal theta. **h** Average theta phase angle of the mean resultant vector from SPC analysis. The theta phase corresponding to the degree value is shown on the right (horizontal axis illustrates voltage of LFP). **i** MRL as a function of lag between prefrontal MUA and hippocampal LFP. Some data was excluded based on lag amplitudes above 100 ms; contributing *N* numbers are stated; statistics identical to **g**. **j** Cross-correlation functions of instantaneous amplitude curves in the connections and frequency bands named at the top of each sub-panel with peak values indicated by a red dot. Statistical indictors in black (WT) or purple (KO) refer to a significant difference of the lag (temporal shift) from 0 ms (Wilcoxon’s signed rank test). **k** Spectra of coherence phase angle along the named connection. Dotted red lines and greek letters indicate analysed frequency bands. **l** Theta-gamma cross-regional PAC for the named directional connections. Solid lines display mean, and shaded area SEM throughout; bars display mean ± SEM throughout. Grey stars in **g**–**l** indicate genotype differences (*t* test in **g**, **i**, **j** and **l**; Watson-Williams test in **h** and **k**). ^#^*p* < 0.1; *p < 0.05; **p < 0.01; ***p ≤ 0.001

An analysis of directional connectivity with parametric GC revealed a confirmatory but much more fine-grained picture with KO-induced aberrations in all four frequency bands depending on the connection and direction (Fig. 3a–c). Most prominently, we found strongly elevated theta range GC in knockouts for all projections departing in either subdivision of the hippocampus. This confirms the hippocampal (as opposed to prefrontal) origin of the theta hyper-connectivity phenotype in *Gria1*-knockout mice that we had postulated before based on the normalization of this phenotype in mice with hippocampal rescue of GluA1 expression [48]. Likewise, beta/gamma dHC➔PFC GC was strongly reduced in knockouts (Fig. 3a), in line with reduced phase synchronization measures (Fig. 2d, j, m, p), while PFC➔dHC beta and gamma GC were even mildly elevated. This again suggests a hippocampal origin of the observed reduced synchrony in this frequency range. The most prominent GC was found in the delta range, with PFC➔d/vHC GC being significantly larger than the delta GC in the opposite direction in both genotypes. Further, genotype-related differences in vHC➔PFC and dHC➔vHC delta GC were found that do not match with the results from the non-directional synchrony metrics (Fig. 2).

In contrast to GC, significantly elevated theta *PDC* in knockouts was only detected in the dHC➔PFC/vHC connections, but not in the vHC➔PFC/dHC projections. And in the beta/gamma ranges, there were virtually no matches between PDC and GC at all regarding genotype-related differences (except for a minority of null-results and trends; Fig. 3a–f). Assessing *SPC* using the mean resultant vector length (MRL) of the vector representing average spike occurrence in theta phase space [21], we found the *opposite* of what would have been assumed from the PDC metric: locking of PFC spikes to *vHC* theta was higher in *Gria1*-knockouts, but phase-locking of PFC spikes to *dHC* theta showed no difference between genotypes (the latter also contrasts with GC and all synchrony measures; Fig. 3g).

Further discrepancies appeared when analysing consistent phase differences (leads and lags) between potentially coupled oscillations in different regions to assess directionality. We investigated two directional measures obtainable from the SPC: the average theta phase of the MRL [20] and analysis of the effect of incremental shifts of the MUA relative to the theta cycle on the MRL [19]. The MRLs of PFC spikes relative to the dHC—but not vHC—theta phase were significantly shifted between genotypes: while they occurred during the rising phase of theta in knockouts, they occurred at its through in wild-type mice (Fig. 3h). Leading of PFC spikes relative to dHC and vHC theta was seen with phase-shifted MRL analysis in knockouts, but no significant difference between genotypes was detectable in this metric (Fig. 3i). The equivalent analysis, but conducted with PFC *LFP* (instead of spikes) using cross-amplitude coupling showed the *opposite*, namely a lead of dHC and vHC theta relative to prefrontal theta in knockouts, and differences between genotypes in both connections (Fig. 3j). In reverse, in the gamma range, PFC led both hippocampal regions exclusively in the knockouts (Fig. 3j), which is not consistent with GC, but—at least for the PFC-vHC connection—with PDC. Lastly, we examined the coherence phase angle. This showed a characteristic ~ 90° shift between the theta, beta and gamma oscillations of the PFC *vis-à-vis* the dHC, particularly in wild-type mice. In contrast to the other directional metrics, significant genotype-related differences were only seen in the gamma range, and they were prominent in the two HC-PFC connections (Fig. 3k).

Finally, dHC- and vHC-*gamma oscillations* were coupled stronger to theta oscillations in PFC and the mutually coupled part of the hippocampus in knockouts (gamma-theta cross-regional PAC; Fig. 3l). However, PFC-gamma to hippocampal theta coupling was even *reduced* in knockouts (Fig. 3l) which contrasts sharply with the results from all other measures.

In summary, while the identification of genotype-related differences in coupling was similar between some measures (especially coherence, PLV, PPC and GC), there was also a considerable lack of redundancy across the different measures of interregional connectivity (see overview in Table 2).

**Table 2:** Pairwise comparison between wild-type and *Gria1*-knockouts

KO vs. WT		Delta			Theta			Beta			Gamma		
		PFC-dHC	PFC-vHC	vHC-dHC	PFC-dHC	PFC-vHC	vHC-dHC	PFC-dHC	PFC-vHC	vHC-dHC	PFC-dHC	PFC-vHC	vHC-dHC
**Average metric**	Coherence				***	***	***				*		
	wPLI	**		*	*	***	**	**	#		*	*	#
	PLV				***	***	***	*			**		
	PPC				***	***	***	*			**		
	GC →				#		*	#			*	#	*
	GC ←		*	*	**	***	*	***			***		#
	PDC →		*			***			*			***	
	PDC ←			**	*	#	**						**
	MRL ←					**							
	MI/PAC →				*	**	*						
	MI/PAC ←				*	#	*						
	Coher. phase			#		#					*	***	#
	CC				***	*	*				*	***	
	MRL-phase ←				*								
	MRL-lag ←												
**Slope metric**	Coherence	***	**	*	**	***	#	**				#	
	wPLI	#				**							
	GC →	**	*		**		*	*				**	
	GC ←		**	**		***	*						*
	MI/PAC →					**							
	MI/PAC ←					*	*						
	CC				*			#					*

Overview over genotype-related statistical comparisons of the data displayed in Figs. 2, 3 (average metric) and 4 (slope metric). GC and PDC results are derived from the Sidak post hoc test after repeated-measures ANOVA across both directions of a connection and genotypes; MRL-phase and coherence-phase angle are compared with the Watson-Williams test [20]; all other *P* values are derived from independent-sample *t* tests. For LFP-based measures (coherence, wPLI, PLV, PPC, GC, PDC), the *P* values in the theta range refer to the peak theta (not mean theta). Arrows in directional measures indicate direction of coupling: → direction labelled in column name (e.g. PFC → dHC in the PFC-dHC column); ← opposite direction. For MI and MRL measures, the region named first corresponds to the region that contributes the theta oscillation to the analysis. *p* ≥ 0.1; ^#^*p* < 0.1; *p < 0.05, **p < 0.01, ***p ≤ 0.001

### Differences in detecting increases of inter-regional coupling over time in *Gria1*-knockouts across measures

**Fig. 4.**
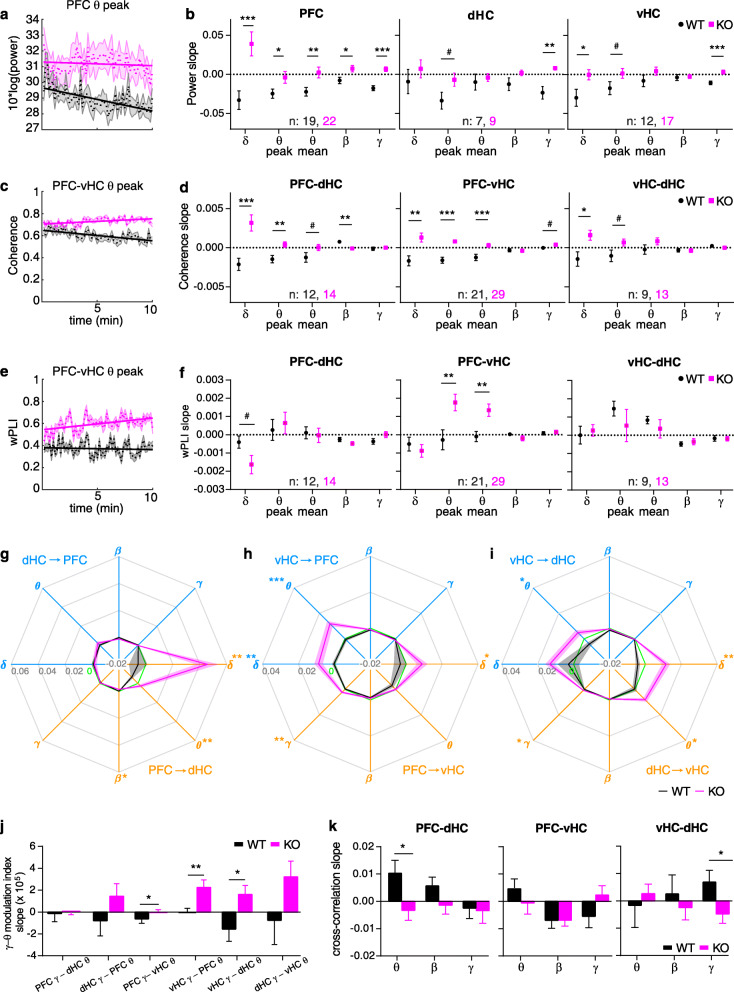
Changes of power and coupling strength over time during the 10 min open-field test. **a**, **c**, **e** Examples of individual measures of power (**a**), coherence (**c**) and wPLI (**e**) as they behave as population average over the 10 min of novelty-induced activity in the open field (dashed line, mean; shaded area, SEM) with linear interpolation between time points overlaid (solid line) to determine the slope as indicator of temporal changes. **b**, **d**, **f** Average slope (temporal change) of power (**b**), coherence (**d**) and wPLI (**f**) in the indicated regions or connections (top of sub-panel) and frequency bands (*x*-axis). **g**, **h**, **i** Slope of GC in the frequency bands indicated by greek letters and along the directional connections identified by the colour (blue: dHC➔PFC (**g**), vHC➔PFC (**h**), vHC➔dHC (**i**); orange: reverse of the before). Statistical indicators in the same colour identify a difference between genotypes (*t* test). **j** Slope of theta-gamma PAC in the stated directional connections. **k** Slope of cross-correlation lags indicating putative changes of temporal shifts of the oscillations in the named frequency bands. Black stars indicate significant differences between genotypes (*t* test), and error bars or shaded regions indicate SEM throughout. ^#^*p* < 0.1; *p < 0.05; **p < 0.01; ***p ≤ 0.001

As a second indicator for redundancy between connectivity measures, we investigated the potential physiological correlates of the characteristic divergence of exploratory drive between the two genotypes over time (Fig. 1b, c). This divergence is likely induced by a failure of spatial short-term habituation in *Gria1*-knockout mice resulting in *increasing* exploration—as opposed to the decreasing activity seen in controls [48, 49]. To allow for an efficient analysis, we captured the change of a given parameter over time in a single number, namely the slope of the linear interpolation across the time series over the 10-min test. We previously found that both local theta power in the dHC and also dHC-PFC theta coherence displayed a characteristic divergence between the groups that mirrored exploratory behaviour [48]. In this novel dataset and analysis, this pattern emerged much more broadly, namely across multiple power and coherence measures in all three connections (compare Fig. 1b, c with Fig. 4a–d). This included local PFC power in all analysed frequency bands and gamma and (at trend-level) theta peak power in the hippocampal regions (Fig. 4a, b). For *coherence*, the KO-related increase in slopes was limited to the delta and theta range and was apparent in the hippocampal-prefrontal connections (confirming our earlier results) and marginally for intra-hippocampal coupling (Fig. 4c, d). In the beta and gamma range, either no group difference occurred or—for PFC-dHC beta coherence—it was even inversed with a higher slope in wild-type mice. Stunningly, this pattern was not reproduced by the *wPLI* analysis (Fig. 4e, f)—even in the one case where the coupling slope was increased in knockouts in *both* metrics (PFC-vHC, theta), the metrics differed in the respect that, in wild-type controls, theta-wPLI remained constant, while theta coherence decreased over time.

*GC* remained largely constant or slightly decreased over time in wild-type mice, irrespective of connection or frequency band (Fig. 4g–i). In *Gria1*^−/−^ mice, in contrast, GC *increased* over time in the delta and theta range in most connections leading to genotype-related differences in the vHC➔PFC (*δ*, *θ*), vHC➔dHC (*θ*), dHC➔vHC (*δ*, *θ*), PFC➔vHC (*δ*, *γ*) and PFC➔dHC (*δ*, *θ*) projections. Thus, except for an isolated match in the vHC➔PFC theta connectivity, the GC metric did not align with the wPLI-based slope assessment but provided a near-perfect match to the coherence slope pattern (Table 2). The latter observation even extends to the one instance of PFC-dHC beta coupling where the slope is higher in wild-type than in KO mice (Fig. 4g–i). The slope of the *gamma-theta PAC* also showed the expected divergence between genotypes in coupling strength along vHC connections, but not in the PFC-dHC connections (Fig. 4j). This pattern matched neither with coherence and GC (as they detected temporal changes in the PFC-dHC connection) nor with wPLI (which detected no changes in the vHC-dHC connection). Likewise, cross-correlational lags did not change in any pattern that resembled the other measures (Fig. 4k). The slopes of MUA-related metrics were not determined because SPC analysis requires a considerable and equal number of spikes (not suitable for short intervals), and PDC and other lag metrics were not further regarded given that they already differed from the other metrics in the first comparison (Fig. 3).

### Lack of redundancy between most coupling measures revealed by bivariate correlation analysis

**Fig. 5.**
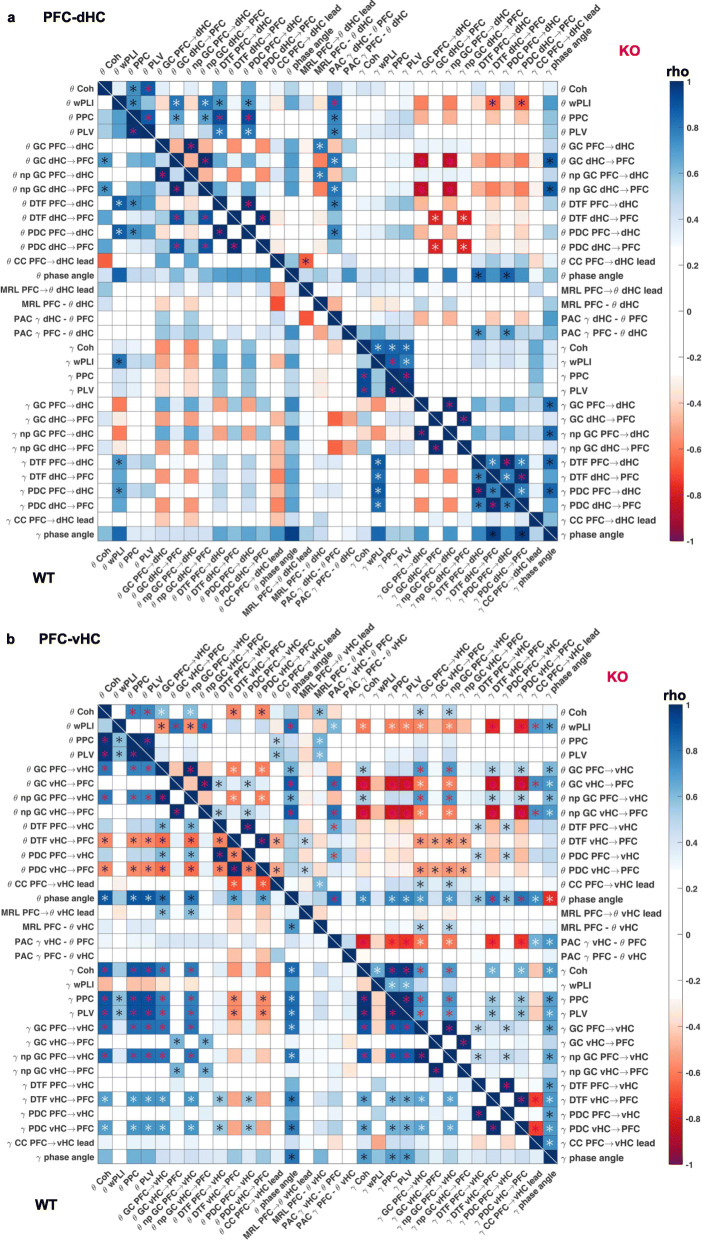
Correlations between individual measures of hippocampal-prefrontal connectivity. **a**, **b** Spearman’s coefficient (rho, colour of squares) and significance (star within squares) of bivariate correlations between individual measures of connectivity in the PFC-dHC (**a**) and PFC-vHC (**b**) connections within KO (top-right) and WT (bottom-left) mice. White stars, *p* < 0.01; purple stars, *p* < 0.001. Theta and gamma metrics are spatially separated, and delta and beta metrics are omitted (see Additional file 1: Table S1 and S2 for pairwise correlations of all metrics analysed in this figure)

**Fig. 6.**
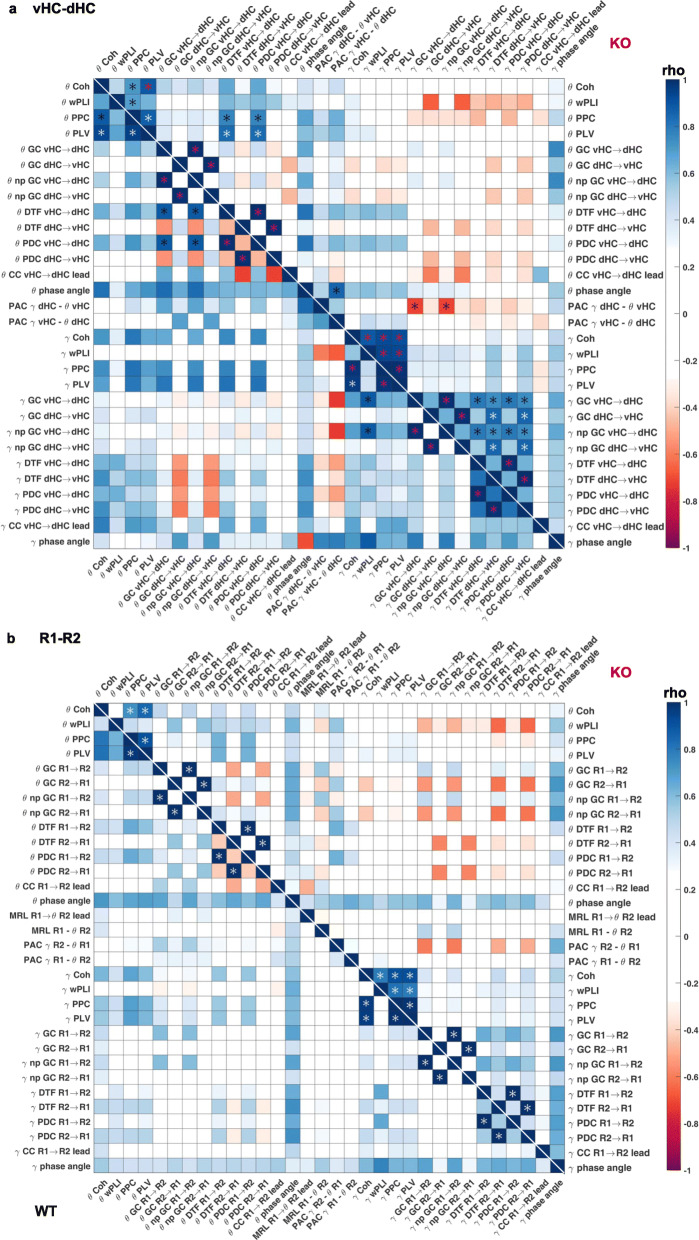
Correlations between individual measures of intra-hippocampal and overall connectivity. **a** Spearman’s coefficient (rho, colour of squares) and significance (star within squares) of bivariate correlations between individual measures of connectivity in the vHC-dHC connection within KO (top-right) and WT (bottom-left) mice. **b** The same display as in **a** but indicating the *average* correlation coefficient across the three connections (Figs. 5a, b and 6a) by the colour of a square and significance only if a significant correlation existed in every one of the three connections. White stars, *p* < 0.01; purple stars, *p* < 0.001. Theta and gamma metrics are spatially separated, and delta and beta metrics are omitted (see Additional file 1: Table S3 and S4 for pairwise correlations of all metrics analysed in this figure)

Given that the above analysis of comparing genotype-related differences across measures ultimately allows only a *qualitative* judgement about the epistemological redundancy of interregional coupling metrics, we supplemented our analysis by a more quantitative analysis in form of bivariate Spearman correlations between pairs of parameters and within genotypes and connections using the average value for each parameter in each electrode pair as a dependent variable. We included all metrics analysed in Figs. 2 and 3 and also partial directed coherence (PDC) and non-parametric Granger causality (npGC). This revealed multiple levels of complexity when analysing the relation between the metrics. On the one hand, at the level of isolated observations, the correlations supported the commonalities between measures already seen with the two prior analyses. For example, PFC-dHC theta coherence correlated strongly with dHC➔PFC theta-GC in wild-types (Fig. 5a). However, this correlation did neither exist in the *knockouts* in the same connection (Fig. 5a) nor in the same genotype but the PFC-*vHC* connection (Fig. 5b). Indeed, PFC-vHC theta coherence did correlate highly with GC in the opposite, i.e. PFC➔vHC, direction but not in the vHC➔PFC direction—and it did so across all frequency bands (Fig. 5b, see Additional file 1: Table S1 and S2 for the full correlation tables in wild-type mice including all metrics and four frequency bands), which was not the case in the other two connections (Figs. 5a and 6a). In general, when carefully examining each pair of metrics, it became apparent that a correlation seen in one genotype and connection would rarely reappear in another one (Fig. 5a, b, 6a).

In order to evaluate this systematically, we calculated the average correlation coefficient for each pair across the three connections and indicated its significance only if it was given in all of them (Fig. 6b). Reassuringly, the three pairs of mathematically closely related metrics showed consistent correlations in each connection and frequency band: PPC and PLV, parametric and non-parametric GC, and PDC and DTF. Beyond that, however, there was not a single pair of distinct metrics that achieved a significant correlation in all three connections in wild-type mice in the theta band, and only two (coherence correlating with PPC and PLV) in the gamma band (Fig. 6b, Additional file 1: Table S4). In *Gria1*-knockouts, the picture was similar, except that, here, coherence correlated significantly with PLV and PPC in both the theta and the gamma bands, and additionally, gamma wPLI correlated with coherence, PPC and PLV, across connections. The latter result contrasts sharply with the absence of such wPLI correlations in wild-type mice, illustrating that some observed correlations may depend on the genotype and are hence not reflecting a priori redundancies.

We further examined the correlations that were not significant in all three connections, but yet achieved a high correlation coefficient on average. In the theta range, *coherence* also correlated strongly with PPC and PLV (average rho ≥ 0.8)—in alignment with our first analysis (Figs. 2 and 3), the correlation result in knockouts, and the gamma band in both genotypes (Fig. 6b)—and with coherence phase angle (average rho > 0.7); further correlations yielded a medium (0.6–0.7) average rho: (a) *coherence phase angle* with PPC, PLV, PDC, DTF, GC and npGC and (b) *PPC/PLV* with wPLI, PDC and DTF. In the *gamma* range, *coherence phase angle* also showed the largest number of medium average correlations with other measures, namely wPLI (average rho = 0.77) and with coherence magnitude, PPC, PLV, PDC, DTF, GC and npGC (average rho 0.6–0.7); the only remaining medium average correlations (0.6–0.7) in the gamma range were wPLI with PDC and DTF (Fig. 6b, Additional file 1: Table S4). Also in knockouts, the *coherence phase angle* showed average medium correlations with most other LFP-based metrics in the theta and gamma range (Fig. 6b). It should be noted that this combined analysis may overlook correlations with directional metrics in case they occur in only one direction. For example, theta GC (and npGC) did actually correlate with theta PDC (and DTF) in each of the three connections but only in one direction each: PFC➔vHC, dHC➔PFC and vHC➔dHC which is difficult to interpret given that we always recorded significant GC and PDC in both directions. Results from the SPC (MRL), PAC and amplitude cross-correlation (lag) analyses did not correlate with any other measure consistently in any genotype. This synopsis largely aligns with the redundancy patterns seen with the two former analyses (Table 2).

### Sensitivity of measures to reference location

The choice of placement site for the reference electrode varies considerably between studies, and both the referencing to the ground screw above the cerebellum (as done for all above analyses) and to the anterior part of the frontal cortex are widely used. In order to investigate the effect of this difference, we recorded a separate reference signal from a frontal reference screw [2, 45] and used it to digitally re-reference all recorded data by subtracting this signal from the recorded LFP traces before re-calculating the local power, coherence, wPLI and GC.

**Fig. 7.**
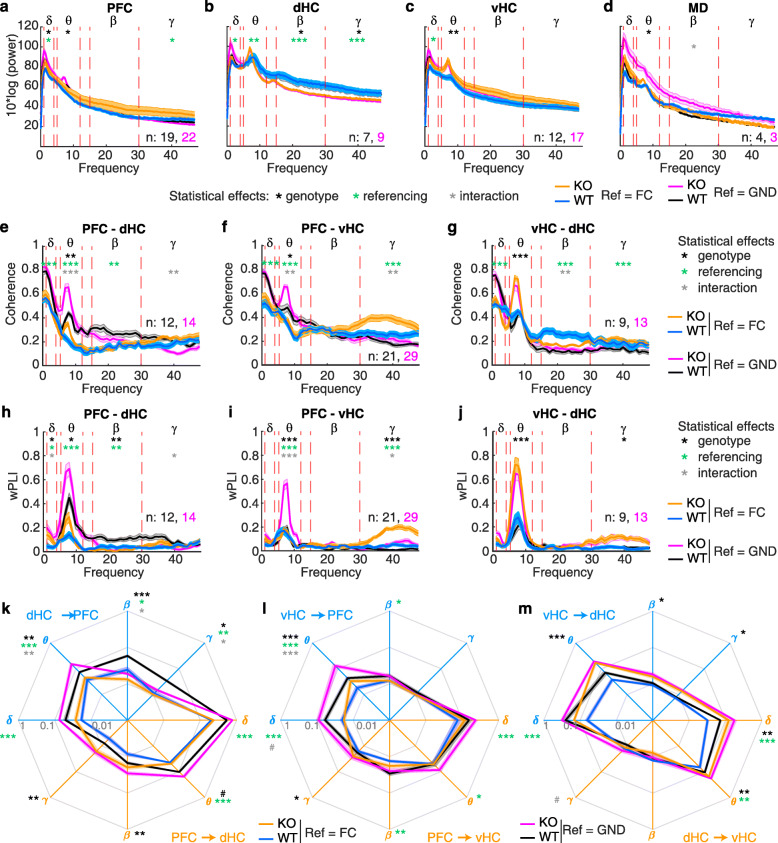
Assessment of the impact of the reference electrode placement on the measurement of power and connectivity. **a**–**j** Spectra for power (**a**–**d**), coherence (**e**–**g**) and wPLI (**h**–**j**) for the regions or connections indicated at the top of each panel, shown for standard referencing to the ground screw above the cerebellum (black, WT; purple, KO; as in Figs. 2 and 3) or digitally re-referencing to the reference screw above the frontal cortex (blue, WT; orange, KO). Red lines indicate the boundaries of the analysed frequency bands named by the greek letters at the top. **k**–**m** GC in the frequency bands indicated by greek letters and along the directional connections identified by the colour (blue: dHC➔PFC (**k**), vHC➔PFC (**l**), vHC➔dHC (**m**); orange: reverse of the before; display as in Fig. 3a–c). Throughout, shaded regions indicate SEM, and stars indicate the results of RM-ANOVA: black, effect of genotype; green, effect of chosen reference; grey, genotype-reference interaction. In the theta range, the statistics for coherence and wPLI refer to peak theta. *p < 0.05; **p < 0.01; ***p ≤ 0.001

Using repeated-measure ANOVAs with the within-subject factor of re-referencing and the between-subject factor of genotype, we found that the location of the reference has quite a substantial influence on the results. There were significant effects of re-referencing on delta power, coherence and GC in all brain regions (except for the MD) and connections, while the effect on wPLI was comparatively minor (but note that delta wPLI is generally very low and entirely different from delta-coherence and GC; Fig. 7a–m). In the theta range, in contrast, re-referencing affected power only in the dHC but strongly impacted coherence, wPLI and GC alike along both hippocampal-prefrontal connections—not only in terms of significant effects of re-referencing, but also in terms of genotype-reference interactions, which indicate that the prior conclusions on theta range connectivity are partly dependent on the position of the reference. In the GC measure, interactions were apparent in the d/vHC➔PFC direction but not in the reverse (Fig. 7k, l). Nevertheless, there were also significant effects of genotype in those connections and measures, suggesting that the fundamental observation of elevated hippocampal-prefrontal theta connectivity in knockouts still holds, especially for the PFC-dHC connection and the GC measure in general (Fig. 7e, f, h, i, k, l). Intra-hippocampal theta connectivity was not much affected by the reference placement, irrespective of measure (Fig. 7g, j, m).

In the higher frequency ranges, the effects were more mixed. Beta power in the dHC and coherence—but only partly wPLI and GC—along its connections were affected by reference placement. In the gamma range, re-referencing impacted power in the PFC and dHC, wPLI in the PFC-d/vHC connections and coherence along all three connections (Fig. 7a–j). In fact, the formerly observed lower PFC-dHC gamma coherence and wPLI in knockouts (Fig. 2d, j) were dependent on the reference placement for detection (interaction effect *only* for coherence and wPLI, Fig. 7e, h). A similar observation holds for the PFC-vHC gamma connectivity which was increased in KOs in the wPLI, but not the coherence measure (Fig. 2e, k). Here again, an interaction indicated that the absence or presence of this difference in the coherence measure depends on the reference location (Fig. 7f), while an effect of genotype is maintained when using wPLI even though an interaction is found in addition (Fig. 7i). The impact of referencing on gamma-GC, in contrast, was limited to the dHC➔PFC projection (Fig. 7k–m).

In summary, a frontal reference screw—as often used when studying prefrontal-hippocampal connectivity [2, 45]—may considerably alter the results obtained for LFP-based measurements of connectivity between the PFC and the hippocampus. Somewhat surprisingly, the wPLI measure does not eliminate this contingency but only reduces it, especially in the beta-gamma range. Referencing effects on GC are particularly visible in the low (delta/theta) frequency range and (as interactions) in the direction from the hippocampus to PFC.

## Discussion

We here examined the level of redundancy and experimental contingencies of the most widely applied measures of interregional directed and non-directed neuronal connectivity that are obtainable with chronically implanted field electrodes in awake rodents. This analysis revealed a surprisingly large absence of redundancies between such metrics and a worrying contingency with respect to the location of the reference electrode. Both findings suggest that the implicitly held belief that experimental results obtained with one metric of connectivity and one configuration for referencing would allow *general* conclusions about aberrations in inter-regional functional connectivity is problematic. Intriguingly, a similar conclusion has been reached by a recent study on connectivity measures applied on human EEG data [50].

While this finding was somewhat expected a priori when regarding metrics of distinct conceptual foundation—e.g. non-directional synchrony vs. measures of causation—the lack of similarity even within the same analytical category is unreckoned. From a conceptual perspective, the result reveal the absence of a concrete empirical counterpart of the rather interchangeably used terms of inter-regional *communication*, *coupling*, *information transfer* or *functional connectivity.* Given these contingencies of a result obtained with any single metric, it is difficult to equate it with the too generic notion of *neuronal communication*.

A particular analytical problem appears to be the lack of benchmarking of the sensitivity, specificity and robustness of the individual measures against a ground truth of actual physiological trans-synaptic activity along anatomically verified connections. Notably, we here, like previous studies, found evidence for significant causal influence not only along the direct anatomical projections—dHC➔vHC [51, 52], vHC➔dHC [52] and vHC➔PFC [52–55]—but also along the PFC-dHC connection that is mediated only *indirectly* via the nucleus reuniens [56, 57], and even in the direction for which no obvious anatomical correlate has been described yet to our knowledge (PFC➔vHC [52]), which complicates the validation and interpretation of the functional connectivity measurements.

In the absence of such benchmarking and while facing considerable logistical limits in applying multiple referencing and metrics for every experiment, our analysis at least qualitatively implies some guidelines to choose the set of coupling metrics suited for a rather comprehensive, yet *non-redundant* analysis of inter-regional communication.

Firstly, we demonstrate that some mathematically related measures *do* actually show a pairwise redundancy and hence do not need to be included into the same analysis, namely PPC and PLV [30], parametric and non-parametric GC (allowing for considerably faster computation by using the non-parametric approach [15]) and PDC and DTF [16, 18].

Secondly, beyond these reliable redundancies, we found further partial redundancies across connections, genotypes and frequencies helping to narrow the list of metrics to include in an analysis further. Most importantly, PPC and PLV also showed considerable overlap with both the magnitude and (to a lesser degree) the phase angle of coherence, and medium average correlations with wPLI, PDC and DTF. In addition, coherence phase angle correlated broadly at a medium average level with coherence amplitude, PDC, DTF, GC and npGC in addition to PLV and PPC. For practical purposes, this suggests that an assessment of two metrics—PPC and coherence phase angle—would be a useful first-pass approach to survey LFP data for possible aberrations in functional connectivity, which can then be followed up with mutually non-redundant directional metrics.

Thirdly, while in such further analysis, GC and PDC (or DTF) may seem particularly attractive metrics given that they deliver a more fine-grained picture of coupling in distinct directions and may be interpreted as indicators of causal influence between two brain regions, it is important to note that they do not yield similar results even though they are sometimes (erroneously [58]) equated. Despite some correlations between PDC (and DTF) with PLV, PPC and coherence phase angle in the correlation analysis (Figs. 5 and 6), there were actually considerable and irresolvable discrepancies between these measures in the genotype comparison (compare Fig. 2 with Fig. 3d–f); for example, genotype-related differences in PFC-dHC gamma-range coupling seen across all measures of synchrony and coherence phase angle were not detected by PDC, while the reverse was true for the vHC-dHC connection. GC, in contrast, did mostly reflect aberrations seen with the synchrony measures and could clarify their directional underpinning (Table 2). Therefore, PDC/DTF and GC may serve as complementary metrics rather than surrogates.

Fourth, spike-phase and phase-amplitude coupling cannot be expected to be equivalent to any of the other parameters and are therefore very useful to include to deliver a different perspective on functional connectivity. While this may have been expected given their distinct biological nature, the degree of absence of redundancy is nevertheless astonishing. It should be noted, however, that the presented SPC analysis using MUA [21] is likely far from optimal given that units cannot be chosen by the movement of the electrodes and not properly sorted. The recording of single-unit activity from moveable electrode bundles or arrays [2, 45] will certainly improve the assessment of SPC and its related directional measures.

Finally, for LFP-based measures, the reference electrode should be placed in a brain structure that is largely separate from the brain regions between which connectivity is studied. A frontal screw may easily obscure phenotypes in prefrontal connectivity as it may pick-up field potential signals from the PFC [29, 59].

## Conclusions

In summary, our analysis calls for a more cautious interpretation of previous findings in the rodent literature on inter-regional coupling (especially when regarding negative results), the need for better benchmarking of individual measures and the necessity to report multiple measures of connectivity in future studies.

## Methods

### Animals

Male and female *Gria1* knockout (*Gria1*^*−/−*^, *Gria1*^tm1Rsp^; MGI:2178057) [60] mice (*N* = 15, 9 males) and wild-type littermate controls (*N* = 12, 8 males) were bred from heterozygous parents. Animals were group-housed in type II long individually ventilated cages (Greenline, Tecniplast, G), enriched with sawdust, sizzle-nest™ and cardboard houses (Datesand, UK) and subjected to a 13-h light/11-h dark cycle. The mice were implanted with electrodes at ca. 9 months of age and were tested in the open-field test ca. 3–5 weeks later to allow recovery from surgery intermittently.

### Surgery

Electrode implantation surgeries under general isoflurane anaesthesia and a broad peri-operative analgesic regime were conducted similarly as previously described for a similar dataset from a distinct cohort [48]. Briefly, single polyimide-insulated tungsten wires of 50 μm diameter (WireTronic Inc., CA, USA) were implanted, with reference to the bregma (in mm), into the PFC (AP + 1.8–1.9, ML 0.3–0.35; 1.8–1.9 below pia), MD (AP − 1.2, ML 0.3, 2.7 below pia), dHC (AP − 1.9–2.0, ML 1.5, 1.4 below pia) and vHC (AP − 3.1–3.2, ML 2.9–3.0, 3.4 mm for single and 3.8–3.9 mm for dual electrodes below pia). In a majority of mice, dual electrodes were used for PFC and vHC, whereby the second electrode was placed about 0.5 mm higher than the stated distance from pia. In later analysis, the data from each electrode was regarded as the unit of observation (*N*), so that a single mouse could contribute up to an *N* = 4 for vHC-PFC connections and up to an *N* = 2 for dHC-vHC, PFC-vHC, MD-PFC and MD-vHC connections. Both hemispheres were implanted at roughly equal proportion. Stainless steel screws (1.2 mm diameter, Precision Technologies, UK) were implanted in the contralateral hemisphere ca. 1 mm from the midline above the cerebellum (AP − 5.5) for ground and above the anterior frontal cortex (AP + 4.0) for additional reference, and were connected with a 120-μm PTFE-insulated stainless steel wire (Advent Research Materials Ltd., UK; Fig. 1a). All electrode wires were connected to pins in a dual-row 6-pin or 8-pin connector (Mill-Max, UK).

To later determine electrode placements *post-mortem*, electrolytic lesions were made after breathing ceased under terminal ketamine/medetomidine anaesthesia. Immediately afterwards, animals were transcardially perfused with PBS followed by 4% paraformaldehyde (PFA)/PBS, and the brains were post-fixed for 24 h in PFA/PBS. Coronal sections of 60 μm were cut on a vibratome in PBS and then washed 3 times in PBS, stained with DAPI and mounted for inspection of lesion sites on an epifluorescence microscope (DM6, Leica).

#### Novelty-induced locomotion and recording

Animals were tethered to enable electrophysiology recordings and then placed into a novel environment consisting of a clear type III plastic cage (length 43 cm, width 22 cm, height 20 cm; Tecniplast) containing clean sawdust. Animals were allowed to explore for 10 min. The animals’ location in the open field was video-tracked with ANY-maze (Stoelting, UK), and the distance travelled was calculated in 20 sec time bins. Prior to testing, a 32-channel RHD2132 headstage (Intan Technologies, CA, USA) was plugged into the implanted connector via a custom-built adaptor that interfaced a 36-pin Omnetics connector (A79022–001, MSA components, G) with another 6-pin or 8-pin Mill-Max connector. The headstage was wired to an *Open-Ephys* acquisition board (https://open-ephys.org, USA; obtained through the Open-EPhys store at Champalimaud, Portugal) via two light-weight flexible SPI-cables (Intan Technologies), daisy-chained through a custom-connected miniature slip-ring (Adafruit, NY, USA). The adaptor was wired so that all signals were referenced to the ground signal obtained from above the contralateral cerebellum, while the signal from the additional frontal reference screw was recorded separately (for later offline re-referencing) like the LFP channels, i.e. also referenced to ground. Using the RHD2132 headstage, the Open-Ephys acquisition board and the Open-EPhys acquisition software, data were amplified and digitized, sampled at 10 kHz and digitally high-pass filtered at 0.1 Hz for the acquisition of raw data (for MUA and GC analysis) and simultaneously band-pass filtered at 0.1–250 Hz (for all remaining analysis of LFP signals).

### Data processing and analysis

All signal analyses were done in MatLab (MathWorks). Data were exported to MatLab and, for all LFP analyses, down-sampled to 1 kHz and analysed with custom-written scripts. To reduce low-frequency drift, signals were first detrended using the *locdetrend* function of the Chronux signal processing toolbox (http://chronux.org/) with 1 s of data and a sliding window of 0.5 s.

#### Spectral analysis

Power and coherence spectra as well as the phase angles were calculated with Chronux routines implemented in the Chronux toolbox using the multi-taper method [61]. Power values were expressed as 10*log_10_ values for all analyses, and the range of frequencies was set from 0.1 to 48 Hz. A bandwidth of 0.2 Hz and a total of 220 tapers were used to calculate power and coherence over the course of the 10 min exploration time. To analyse the temporal development, power and coherence were also calculated in 10-s bins using a bandwidth of 1 Hz and 19 tapers.

#### Weighted phase lag index

To address the issue of volume conduction, we calculated the weighted Phase Lag Index (wPLI) [5] using the routines implemented in the FieldTrip toolbox [62]. The 10-min exploration time was divided into non-overlapping 1-s bins and padded to the next power of two. The complex cross-spectrum was computed using a Hann taper with a spectral smoothing of 0.5 Hz. For temporal analysis, wPLI was averaged for each minute of the 10-min period using the same spectral parameters.

#### Phase-locking value and pairwise-phase consistency

Phase-locking was assessed using two of the most widely used metrices, namely the phase-locking value (PLV) [6] and pairwise-phase consistency (PPC) [7]. Both were calculated using routines implemented in the FieldTrip toolbox [62]. The 10-min exploration time was divided into non-overlapping 1-s bins and padded to the next power of two. The complex cross-spectrum was computed using a Hann taper with a spectral smoothing of 0.5 Hz.

#### Phase-amplitude coupling

Cross-frequency coupling (CFC, [36]) was assessed using the measure of phase-amplitude coupling (PAC), the statistical relationship between the phase of a low-frequency and the amplitude of a high-frequency component, in a cross-regional analysis [22, 23]. The 10-min recording was split into 1-min bins during which the PAC was calculated using the Modulation Index (MI, [23, 63]). Briefly, time-series data was first band-pass filtered in the desired frequency range, followed by a Hilbert transform using the MatLab function *hilbert* which calculates the real and imaginary part of the signal to obtain the instantaneous amplitude and phase at any given time point. Theta phases were binned into eighteen 20° intervals, and the mean gamma amplitude was calculated in each phase bin. The distribution across bins was assessed using the Kullback-Leibler divergence [64] and normalized between 0 and 1. The MI is close to zero if the mean gamma amplitude is uniformly distributed over the theta phases and close to one if the mean gamma amplitude is exceptionally higher within one phase bin [23].

#### Cross-correlation of instantaneous LFP amplitudes

To determine whether one signal was leading or lagging the other, amplitude cross-correlations of instantaneous amplitudes of LFP oscillations between all brain regions were performed [10]. The 10-min period was divided into 1- s bins with a 95% overlap. First, the two signals were band-pass filtered in the respective frequency range; the Hilbert transform was computed using the MatLab function *hilbert* to calculate the instantaneous amplitude and the envelope of the signal. The mean amplitude was subtracted, and the cross-correlation between the amplitudes of the two signals was calculated with the MatLab function *xcorr* over lags ranging from − 100 to + 100 ms; the lag at which cross-correlation peaked was determined [10]. While lags below − 100 ms or above 100 ms would have led to the exclusion of the respective data point [65], no instances of such lags were found in our dataset. To determine if the obtained lags or leads significantly differed from zero, Wilcoxon’s signed rank tests were performed.

#### Granger causality

Parametric Granger causality (GC) was calculated using the MVGC-toolbox [66]. GC mainly applies to stationary signals which means that the variances are not excessively changing over time [13, 67]. Therefore, the 10-min period was divided into 1-min bins and the in-built trial averaging function was used to calculate GC in non-overlapping 10-s sections to ensure reasonable stationarity [68–70]. The 1-min bins were used for the analysis of GC over time and then averaged to obtain a GC value for the whole 10-min testing period. Raw LFP data was sampled down to 250 Hz to ensure a reasonable model order for autoregressive modelling [14, 66, 71]. The model order was obtained using the Bayesian Information Criterion (BIC, [72]) as it was shown to provide the best fit to electrophysiological data [66]. The model order was fixed to 27 across all animals and trials to obtain comparable results [73]. Non-prefiltered data were used because empirical analyses have shown that filtering time-series data increases the VAR model order and leads to high variances making it unsuitable for GC analysis [71]. To obtain GC values for specific frequency bands, we first computed GC up to the Nyquist frequency and then integrated over the desired frequency range [71]. A permutation procedure implemented in the MVGC-toolbox was performed to test the null hypothesis that values obtained by GC estimation occurred by chance [13, 66]. Non-parametric Granger causality (npGC), directed transfer function (DTF) and partial directed coherence (PDC) were calculated using the FieldTrip toolbox [62]. The same temporal configurations were used as described above for parametric GC, and raw LFP data was sampled down to 250 Hz as well. Instead of deriving the noise covariance matrix and transfer function by autoregressive modelling (as done for parametric GC), these were obtained by applying Wilson’s spectral matrix factorization to complex Fourier spectra. This non-parametric approach was shown to be better at capturing all spectral features, less error-prone because no model order had to be chosen and computationally faster [15, 35].

#### Spike-phase coupling

Multi-unit activity was extracted by high-pass filtering the raw signal above 800 Hz and applying a threshold at 3.5 standard deviations from the mean. Spikes were excluded, if the threshold exceeding was longer than 2 ms, and if spikes occurred within 1 ms form each other. LFP of the second brain region was filtered between 5 and 12 Hz using the *eegfilt*—function of the EEGLAB-toolbox [74]. To account for speed-dependent waveform asymmetry in the theta oscillation, the theta phase was defined by linear interpolation between troughs of consecutive cycles [75, 76]. Only periods in which the theta amplitude was above 0.25 standard deviations of its mean were included to ensure sufficient theta oscillations and prevent spurious phase determination. The number of spikes was fixed to 1000 for each recording to prevent spuriously high MRL values and fluctuations in the firing rate. Each spike was assigned a theta phase, and the mean resultant vector length (MRL) was calculated as an indicator for the strength of coupling using the CircStat-Toolbox [2, 77]. The MRL gets close to one when the spikes are concentrated around a certain phase of the theta oscillation and approaches zero when they are uniformly distributed. Additionally, the phase angles of the mean resultant vector were used to quantify the differences in phase angles between genotypes, which were statistically assessed with the Watson-Williams test for two samples [20, 77].

To determine the directionality between multiunit activity and theta oscillations, phase-locking was calculated for 50 different temporal offsets ranging from − 100 to + 100 ms in steps of 4 ms. If the MRL peaked at a positive offset, spikes were most strongly locked to the next theta cycle, suggesting that spiking activity drives theta [19]. Wilcoxon’s signed rank test was applied to determine if the lag or lead was significantly different from zero.

### Statistics

Genotype-related differences within the same metric and frequency range were assessed by independent-sample *t* test or, in the case of GC (Fig. 3), by Sidak paired post hoc tests conducted after a significant effect of genotype or interaction in the prior repeated-measures (RM) ANOVA. For circular data (spike and coherence phase angles) the Watson-Williams two-sample test was used to assess genotype-related differences. A *p* value < 0.05 was used as an indicator for statistical significance; no further correction for multiple comparisons were applied, given that we aimed to emulate the situation that only a single measure is used to characterize connectivity, and false negatives were to be avoided given the analytical goal of detecting redundancies between metrics. Bivariate correlations were calculated using Spearman’s rho. To detect correlations between circular and circular and between circular and linear data, we used circular-circular correlation and circular-linear correlation as implemented in [77]. Variability in the data is displayed as standard error of the mean (SEM) throughout.

## Supplementary Information

**Additional file 1**: Contains four tables, **Table S1**-**S4**, that state the results of pairwise Spearman correlations between all metrics analysed in this study for a given connection. The first number in each cell states Spearman’s rho, the second number its *p*-value. Significant correlations are highlighted in green. See the ‘Info’ sheet of the file for further information, including *n*-numbers. **Table S1.** Correlations of connectivity metrics along the PFC-dHC connection, corresponding to main Figure 5a. **Table S2.** Correlations of connectivity metrics along the PFC-vHC connection, corresponding to main Figure 5b. **Table S3.** Correlations of connectivity metrics along the vHC-dHC connection, corresponding to main Figure 6a. **Table S4.** Average correlations of connectivity metrics along all three connections, corresponding to main Figure 6b. Each cell lists the average Spearman’s rho, followed by an indicator of a significant correlation in all three connections (0) or lack of it (1).

## Data Availability

The electrophysiological datasets used and analysed in the current study are deposited under https://gin.g-node.org/KaetzelLab/GluA1-KO_LFP_data [78]. All MatLab® analysis scripts are available on https://github.com/KaetzelLab/LFP_analysis [79].
